# Orthopedic disease classification based on breadth-first search algorithm

**DOI:** 10.1038/s41598-024-73559-6

**Published:** 2024-10-08

**Authors:** Ahmed M. Elshewey, Ahmed M. Osman

**Affiliations:** 1 Department of Computer Science, Faculty of Computers and Information, Suez University, P.O.Box:43221, Suez, Egypt; 2 Department of Information Systems, Faculty of Computers and Information, Suez University, P.O.Box:43221, Suez, Egypt

**Keywords:** Orthopedics, Hybrid RF model, BFS-RF, ML, Healthcare, Orthopedic disease classification, Computational biology and bioinformatics, Mathematics and computing

## Abstract

Orthopedic diseases are widespread worldwide, impacting the body’s musculoskeletal system, particularly those involving bones or hips. They have the potential to cause discomfort and impair functionality. This paper aims to address the lack of supplementary diagnostics in orthopedics and improve the method of diagnosing orthopedic diseases. The study uses binary breadth-first search (BBFS), binary particle swarm optimization (BPSO), binary grey wolf optimizer (BGWO), and binary whale optimization algorithm (BWAO) for feature selections, and the BBFS makes an average error of 47.29% less than others. Then we apply six machine learning models, i.e., RF, SGD, NBC, DC, QDA, and ET. The dataset used contains 310 instances and six distinct features. Through experimentation, the RF model led to optimal outcomes during comparison to the remaining models, with an accuracy of 91.4%. The parameters of the RF model were optimized using four optimization algorithms: BFS, PSO, WAO, and GWO. To check how well the optimized RF works on the dataset, this paper uses prediction evaluation metrics such as accuracy, sensitivity, specificity, F-score, and the AUC curve. The results showed that the BFS-RF can improve the performance of the original classifier compared with others with 99.41% accuracy.

**Supplementary Information:**

The online version contains supplementary material available at 10.1038/s41598-024-73559-6.

## Introduction

The significant obstacles faced by those suffering from spinal disc illnesses are beyond the average individual’s comprehension. Orthopedic problems happen frequently, not just among athletes but also among everyone else, regardless of any prior history of muscle and joint discomfort. Herniation, where organs protrude through muscle and tissue holes, can cause acute discomfort and an inability to move, significantly impacting an individual’s everyday life. Advanced technologies are developing to alleviate the suffering of people with orthopedic problems, but early prevention is considered more effective and beneficial in treating the problem and ensuring a full return to normal^1^.

### Problem statement

Machine Learning (ML) is an emerging method for effectively predicting many diseases at an early stage, using medical information about people with those diseases. A machine-learning-based classification model can provide significant support to those who live in remote areas with limited access to skilled medical professionals^2^. Researchers are progressively employing strategies of early detection, prevention, and treatment for disorders that impact the muscles and bones in the body. Nevertheless, the absence of improved technologies and early intervention results in several individuals experiencing discomfort in their muscles and developing severe illnesses.

### Objectives

The objective of this study is to offer valuable knowledge regarding the early prediction of orthopedic problems, with the goal of averting the progression of more severe diseases and limiting the transmission of diseases among patients.

### Contribution

An RF-optimized model based on BFS was developed for classifying orthopedic disease abnormal and normal people in this research. The dataset used contains 310 instances and six distinct features, and the target class was binary, with 1 indicating normal and 0 indicating abnormal. The data collecting phase guarantees the data is both comprehensive and well-structured. Effective management of noisy missing values is a substantial challenge, frequently arising from mistakes made during data recording. Two approaches are utilized: eliminating missing samples or rectifying null values by replacing them with the average value for each characteristic. The study uses binary breadth-first search (BBFS), binary particle swarm optimization (BPSO), binary grey wolf optimizer (BGWO), and binary whale optimization algorithm (BWAO) for feature selections, and the BBFS makes an average error of 47.29%. Then we apply six machine learning models, i.e., random forest (RF) classifier, stochastic gradient descent (SGD) classifier, Naïve Bayesian classifier (NBC), dummy classifier (DC), quadratic discriminant analysis (QDA) classifier, and extra trees (ET) classifier. Through experimentation, the RF model led to optimal outcomes during comparison to the remaining models. The parameters of the RF model were optimized using four optimization algorithms: BFS, PSO, WAO, and GWO. The dataset used contains 310 instances and six distinct features. The results showed that the developed BFS-RF can improve the performance of the original classifier compared with other hybrid models. It was found that the BFS-RF performs better on the dataset, with an accuracy of 99.41%.

### Paper organization

The research article is structured as follows: the second section outlines previous studies regarding diagnosing orthopedic diseases. The third section of the paper introduces the Breadth-First Search (BFS) method for optimizing the Random Forest (RF) model for classifying orthopedic diseases. The fourth section assesses the optimized RF model and compares it with different machine learning (ML) models using default parameters for classifying orthopedic diseases. In the end, the conclusion and future research directions.

## Related works

The application of modern technology in the field of traditional medical care led to the era of intelligent medicine, thanks to the rapid progress of science and technology. Artificial intelligence (AI) is a highly significant technology that has greatly facilitated contemporary treatment^3^. The majority of orthopedic patients seeking medical care in the hospital’s urgent care center had critical conditions, including open, painful fractures, joint dislocations, or multiple system-integrated injuries. Nevertheless, the overall congestion in an urgent care center, along with inadequate medical resources and overwhelmed staff, often leads to delayed care for patients and emerging medical care problems^4^.

Yao et al.^5^ constructed a model based on deep learning (DL) to prioritize patients, using medical records from 864,043 emergency department patients over a span of 5 years. The training of the model involved the utilization of convolutional neural networks (CNN), recurrent neural networks (RNN), and attention mechanisms. The model exhibited exceptional precision as well as efficacy in forecasting death and admission, surpassing traditional approaches by 0.3–0.5%.

Raita et al.^6^ constructed four machine learning models with medical data from 135,470 patients in an emergency room. The models were trained using triage data as predictors. Following supervised training, the models were evaluated to forecast potential clinical outcomes, such as hospitalization, critical care, and in-hospital mortality. All four algorithms outperformed traditional emergency severity index (ESI) in predicting outcomes, improving clinical triage decision-making, delivering superior care, and maximizing resource allocation for injured patients.

Kwon et al.^7^ utilizes clinical information as a predictor factor to forecast in-hospital mortality, critical care, and admission of emergency department patients. The findings indicated that the AUROC (Area Under the Receiver Operating Characteristic) and P-R (Precision-Recall) curves achieved values of 0.93 and 0.26, respectively, surpassing alternative measures such as the Korean triage and acuity score, modified early warning score, logistic regression, and random forest. The implementation of a machine learning algorithm, specifically XGBoost, for triage and acuity scoring has the potential to significantly improve the accuracy of predictions. This could result in a higher level of certainty for the treatment and care of injured patients in the emergency department.

Wang et al.^8^ created and trained a deep neural network (DL) structure called WrisNet, utilizing a dataset of 4346 hand X-rays. For pre-processing and augmentation, the framework used gray scaling and data augmentation techniques. At a joint over combination value of 0.5, the network successfully obtained an average precision (AP) of 0.55 in hairline finger detection. This is an improvement of at least 0.05 compared to other frameworks.

Pranata et al.^9^ performed a comparison between two deep learning architectures, ResNet and VGG, in order to determine their effectiveness in identifying calcaneus fractures on CT scans. In addition, they utilized the SURF approach, canny edge detection, and contour tracing. ResNet had equivalent accuracy to VGG but exhibited superior performance when used with a deep neural network (DNN) architecture.

Cheng et al.^10^ constructed PelviXNet, a sophisticated deep learning network that was trained using 5204 pelvic X-rays. The data underwent cropping, resizing, and augmentation using random translation, rescaling, flipping, and rotation. PelviXNet, following its training, obtained an AUROC (Area Under the Receiver Operating Characteristic) value of 0.97 when tested on a clinical population set consisting of 1888 pelvic X-rays. The accuracy, sensitivity, and specificity of PelviXNet were measured at 0.92, 0.90, and 0.93, respectively.

Yaqoob et al.^11^ introduced SCACSA as a new method for classifying cancer types through gene expression. They tested SCACSA which combines SCA and CSA on well-known datasets for breast cancer and found that it achieved superior accuracy compared to other methods. Joshi and Aziz^12^ proposed CSSMO and SMOCS which are a new hybrid methods for classifying diseases using deep learning. SMOCS is an effective part of the system which acts as a feature selection tool to identify the most relevant genes from a dataset. The genes which are selected are fed into a deep learning model for classification. They tested their approach on six popular datasets and showed that it outperforms not only other deep learning methods but also traditional machine learning models^13^.

Mahto et al.^14^ introduced CSSMO that is a hybrid model for FS and DL for disease classification. it is first approach that identifies the most relevant genes (features) from a dataset and then uses deep learning to categorize those genes into disease classes. They tested CSSMO on eight datasets according to cancer and approved that is more accurate than existing machine learning and deep learning methods for these tasks. Saxena et al.^15^ compared the performance of a new method called MPCA to several existing optimization algorithms (WOA, SCA, GWO, DE, and ABC) for classification tasks. They assessed these methods based on accuracy, features, and fitness values. To ensure a fair comparison, they analyzed the results using box plots and examined how quickly each method converged on an optimal solution. They studied how to choose the best features for predicting COVID-19 using the MPCA method. They compared MPCA to other algorithms and found that MPCA performed the best in terms of accuracy. They also created variations of MPCA and compared them to the original version, finding that the original MPCA performed well overall.

Neggaz et al.^16^ proposed MRFOSCA for feature selection. This method combines MRFO and SCA algorithms. They tested MRFOSCA on datasets from a public repository and compared it to other existing techniques. They evaluated five different criteria of performance, including how many features were chosen. Notably, some datasets had a very large number of features to begin with. Their findings showed that MRFOSCA consistently achieved better accuracy on classification tasks than other methods, even recent ones. Additionally, MRFOSCA tended to pick the fewest features while still reaching the best accuracy, and it did this in a reasonable amount of time for most datasets.

Houssein et al.^17^ introduced a new method, MRFO-SVM, for automatically classifying ECG signals. MRFO-SVM uses a technique called MRFO to choose the most important attributes and fine-tune the SVM algorithm to enhance classification accuracy. The approach works in three steps: first, it preprocesses the ECG signal, then it extracts features using a new method described in the paper, and finally, it optimizes and classifies the features using MRFO-SVM. Compared to other methods, MRFO-SVM showed good results, suggesting it could be a useful tool for diagnosing heart disease based on ECG signals.

The study by Hashim et al.^18^ introduced a new method called mHGS to improve feature selection (FS). mHGS builds on the Hunger Games Search algorithm but with a specifically improved local escaping operator (LEO). This LEO helps avoid getting stuck on unimportant solutions (local optima), converge on better solutions faster, and explore the search space more effectively. They applied mHGS to a real-world issue: analyzing voice recordings to help diagnose Parkinson’s disease. They compared mHGS to other popular FS methods. Overall, the results showed that mHGS achieves better performance than existing techniques in terms of accuracy, the number of features chosen, and other key metrics. This suggests mHGS is a promising new tool for FS tasks.

The research by Hussain et al.^19^ introduced a new method called SCHHO and evaluated its effectiveness on FS. SCHHO was tested on 16 datasets with varying dimensions and covered over 15,000 features. It achieved feature reduction up to 87% while maintaining high accuracy up to 92%. This performance surpassed other population-based optimization methods and recent hybrid techniques designed for feature selection.

Table 1 compares previous medical categorization feature selection research publications to the suggested technique.

**Table 1:** Some related works focus on feature selection and medical classification.

References	Major contribution	Methodology	Data	Enhancement
Yao et al.^5^	Patient prioritization in emergency department	Deep learning (DL) with CNN, RNN, attention	864,043 patients over 5 years	0.3–0.5% improvement in predicting death and admission
Raita et al.^6^	Emergency department triage	Machine learning models with triage data	135,470 patients	Outperformed traditional ESI in predicting hospitalization, critical care, and mortality
Kwon et al.^7^	Emergency department patient outcome prediction	XGBoost with clinical data	Clinical data from the Korean National Emergency Department Information System (NEDIS)	Achieved high AUROC (0.93) and P-R (0.26) for predicting mortality, critical care, and admission
Wang et al.^8^	Hand X-ray hairline finger detection	Deep neural network (WrisNet)	4346 hand X-rays	0.05 improvement in average precision (AP)
Pranata et al.^9^	Calcaneus fracture detection in CT scans	Deep learning (ResNet vs. VGG) with SURF, canny edge detection, and contour tracing	Computed tomography (CT) images for calcaneus fractures	ResNet performed similar to VGG in accuracy but better with DNN
Cheng et al.^10^	Pelvic X-ray analysis	Deep learning network (PelviXNet)	5204 pelvic X-rays	Achieved AUROC of 0.97, accuracy of 0.92, sensitivity of 0.90, and specificity of 0.93
Yaqoob et al.^11^	Cancer classification with gene expression data	SCACSA (combines SCA & CSA algorithms)	Breast cancer datasets	Achieved superior accuracy compared to previous methods
Joshi and Aziz^12^	Disease classification	Hybrid method (CSSMO & SMOCS) with deep learning	6 benchmark datasets	Outperformed deep learning and traditional machine learning models
Mahto et al.^14^	Disease classification	Feature selection (CSSMO) with deep learning	8 cancer datasets	More accurate than existing machine learning and deep learning methods
Saxena et al.^15^	Feature selection for COVID-19 prediction	MPCA	Comparative analysis on standard datasets	Achieved high accuracy, used fewer features, and converged quickly
Neggaz et al.^16^	Feature selection	MRFOSCA (combines MRFO & SCA algorithms)	Public repository datasets	Achieved better accuracy, used fewer features, and worked well on large datasets
Houssein et al.^17^	ECG signal classification for heart disease diagnosis	MRFO-SVM	MIT-BIH database	Achieved good results in classifying ECG signals
Hashim et al.^18^	Feature selection for Parkinson’s disease diagnosis	mHGS (enhanced Hunger Games Search algorithm)	Voice recordings	Achieved better accuracy, used fewer features, and worked well for real-world tasks
Hussain et al.^19^	Feature selection	SCHHO	16 datasets with varying dimensions	Reduced features by up to 87% while maintaining high accuracy

## Materials and methods

Orthopedic diseases are widespread worldwide, impacting the body’s musculoskeletal system, particularly those involving bones or hips. They have the potential to cause discomfort and impair functionality, making routine daily tasks tough. In this section, we described the dataset that was utilized in this study, followed by the preprocessing steps applied to the data. Subsequently, the feature selection where the approaches used to extract and select the most relevant features from the data, followed by the proposed method in detail. Subsequently, the layout of machine learning (ML) models that were used in this study to evaluate our proposed model, then fitness function describes the function used to guide the optimization of the proposed model.

### Dataset

The dataset utilized in the study is available online^20^. The dataset is depicted as the following: 6 attributes and 310 instances. The dataset includes six biomechanical properties for each patient, which are calculated based on the form and orientation of the pelvis and lumbar spine. These attributes include pelvic incidence, pelvic tilt, lumbar lordosis angle, sacral slope, and pelvic radius. Each characteristic has been utilized as a column in the dataset, which has been transformed into a Comma Separated Values (CSV) file.

#### Data preprocessing

This stage is utilized to guarantee that the used data is complete and organized.

#### Clean null values

Managing noisy missing values is a considerable problem that will likely take considerable time. Null values commonly result from errors that appear during data collection, such as leaving an empty place for diagnostic attributes that don’t apply^21^. NaN, or null indicators, commonly represent missing values. Deleting duplicate rows and columns is essential. Therefore, we suggest two strategies to resolve this issue. One approach is to remove the samples with missing values; however, this may result in the loss of important data. The alternative approach is to impute null by substituting these values with the mean value for each attribute. We replaced the null with a known value from the dataset to preserve the majority of the data’s meaning. Eliminating data that is absent from the dataset would limit its dimensions, perhaps leading to inaccurate analysis. Conversely, retaining missing data could cause abnormalities in the variable distribution. The study uses the K-Nearest Neighbor (KNN) technique for null imputation^22^, identifying ‘k’ samples near the dataset through the Euclidean distance. The average of the ‘k’ neighbors is used to impute null. This method is useful at outliers and requires less computational time, with a value of ‘k’ of 10.

#### Normalization

Normalization is an essential preprocessing step, particularly when dealing with approaches that are impacted by scaled features, such as support vector machines and K-nearest neighbors. We normalized our numerical features using the StandardScaler from scikit-learn, which adjusted them to have a mean of zero and a standard deviation of one. This technique standardizes the scale of all features to prevent any one characteristic from overriding the others during model training.

1 $$\:z=\frac{x-\mu\:}{{\upsigma\:}}$$

The formula represents the transformation of input data x into standard scores, allowing for a mean $\:\varvec{\mu\:}$ of zero and a standard deviation $\:{\upsigma\:}$ of one, by transforming the training samples into standard scores.

#### Feature selection

Feature extraction improves in identifying the efficient attributes for the classifier to learn from the depiction. Prior to evaluating the performance of a model, hyper-parameter optimization allows for precise adjustments. Table 2 shows the performance of BBFS algorithm compared with another algorithms.

**Table 2:** The performance of BBFS algorithm compared with another algorithms.

	BBFS	BPSO	BWAO	BGWO
Average error	0.4729	0.5142	0.5483	0.5838
Average select size	0.4951	0.5246	0.5516	0.6054
Mean fitness	0.4621	0.4851	0.4876	0.4974
Best fitness	0.3861	0.4273	0.4484	0.4595
Standard deviation fitness	0.2816	0.2957	0.3068	0.3147

**Fig. 1:**
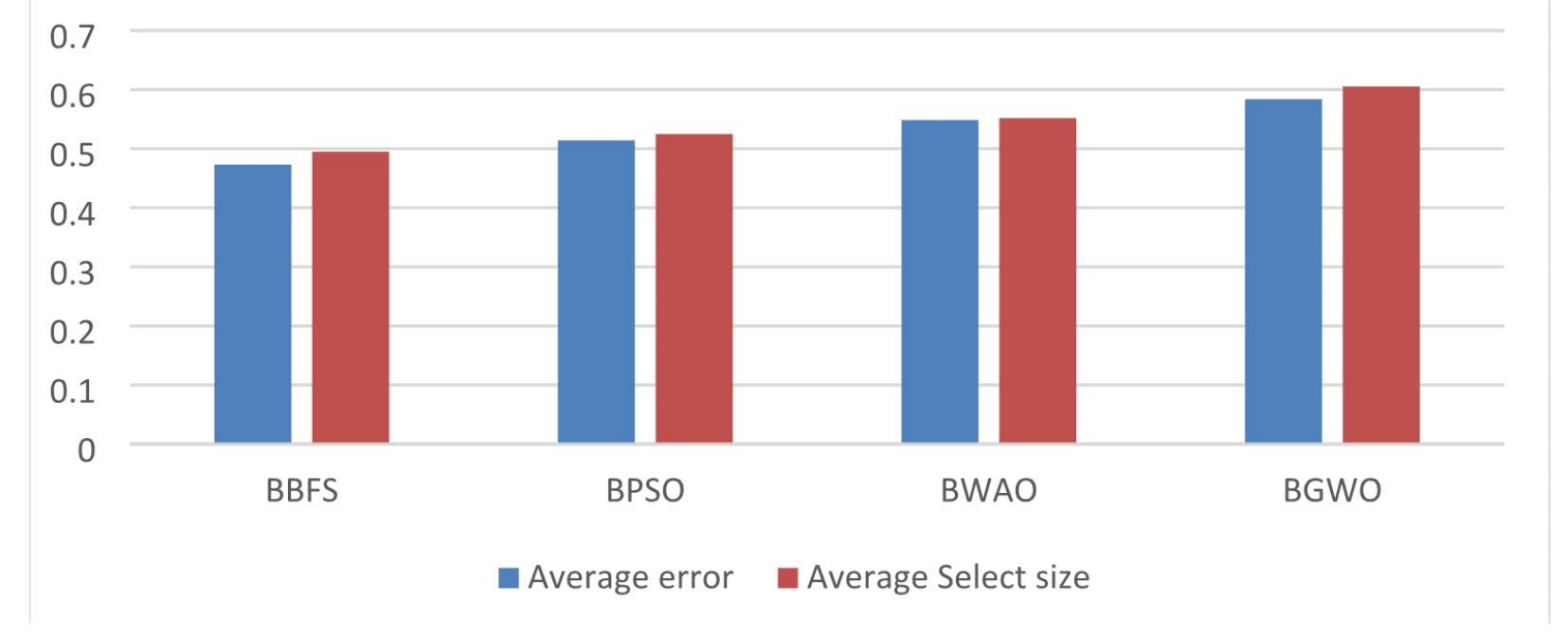
Average error and select size of BBFS compared with other optimizers.

The study uses BBFS, BPSO, BGWO, and BWAO for feature selections, and the BBFS makes an average less error as shown in Fig. 1. Details of BBFS is explained in Binary Breadth-First Search section.

2 $$\:Average\:Error\:\left(AE\right)=\frac{1}{n}\sum\:_{i=1}^{n}{e}_{i}$$

where $\:{e}_{i}$ = $\:f\left({x}_{i}\right)-f\left({x}_{optimal}\right)$ if you’re comparing the function value at the current iteration $\:f\left({x}_{i}\right)$ to the optimal function value $\:f\left({x}_{optimal}\right)$. $\:n$ is the number of iterations or data points.

The entire dataset was split into two separate parts, each of which was used as input for our classification study. Table 3 shows the statistical investigation of the utilized dataset.

**Table 3:** Statistical investigation of the utilized dataset attributes.

Features	num	average	STD	Min	50%	max
Pelvic_incidence	310.0	60.496653	17.236520	26.147921	58.691038	129.834041
Pelvic_tilt numeric	310.0	17.542822	10.008330	− 6.554948	16.357689	49.431864
Lumbar_lordosis_angle	310.0	51.930930	18.554064	14.000000	49.562398	125.742385
Sacral_slope	310.0	42.953831	13.423102	13.366931	42.404912	121.429566
Pelvic_radius	310.0	117.920655	13.317377	70.082575	118.268178	163.071041
Degree_spondylolisthesis	310.0	26.296694	37.559027	− 11.058179	11.767934	418.543082
Class	310.0	0.677419	0.468220	0.000000	1.000000	1.000000

**Fig. 2:**
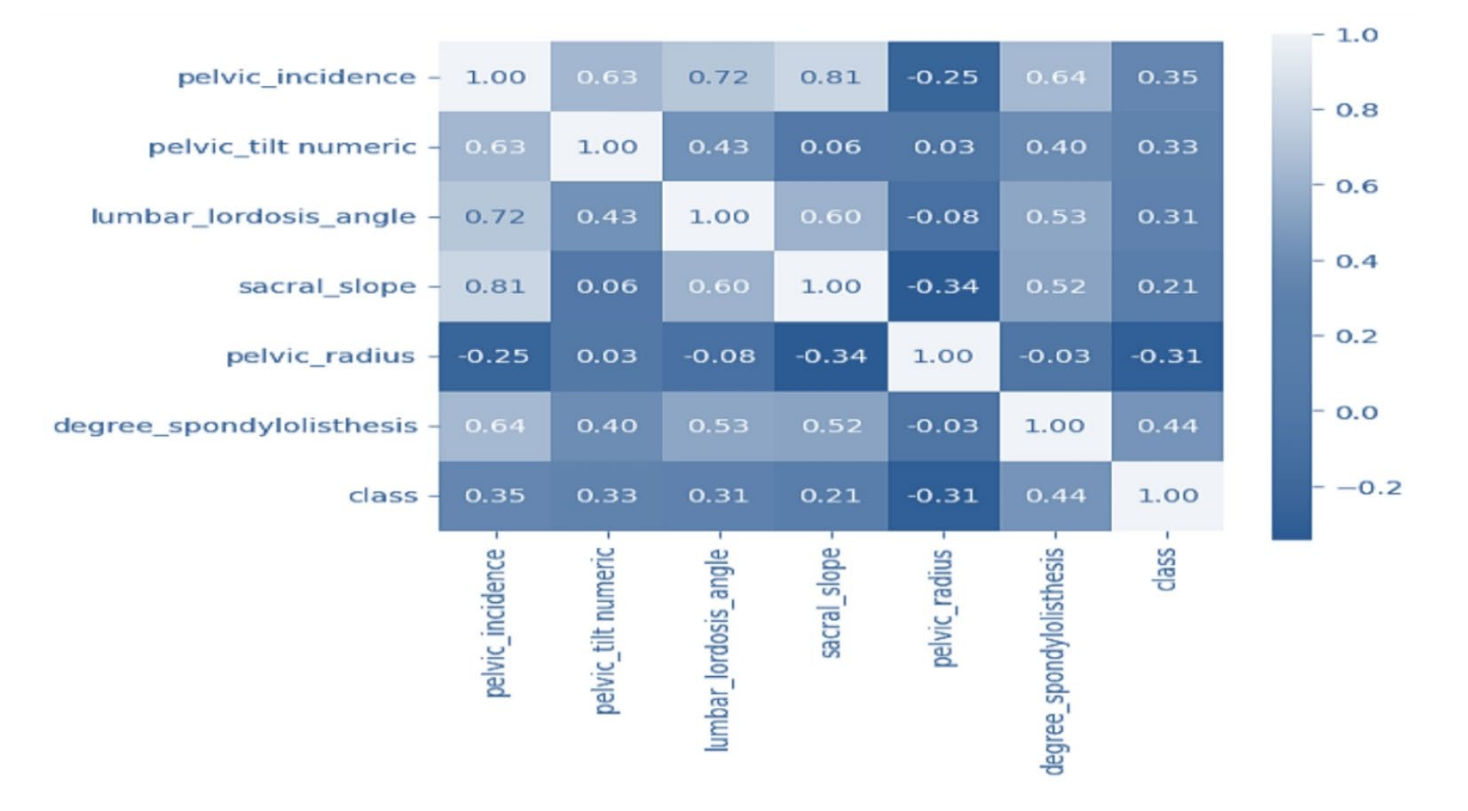
Heatmap investigation for utilized dataset attributes.

**Fig. 3:**
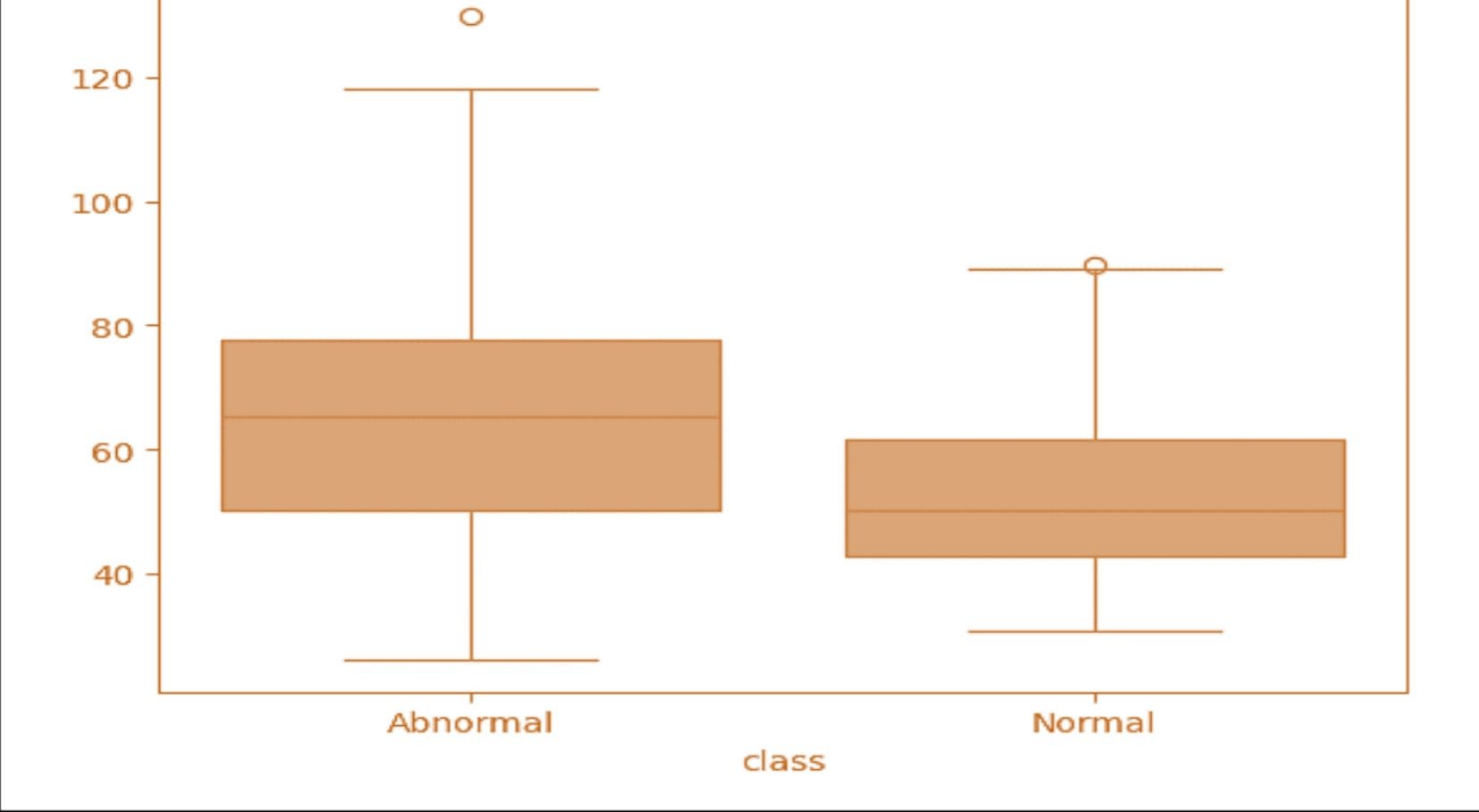
Boxplot visualization of the target attributes.

Figure 2 displays the heatmap investigation of the dataset features. Heatmap analytics is a frequently utilized method that visualizes the correlation between variables in a dataset. We use it to determine the strength and weakness of the connections between variables and to identify the correlation among them. Both bars in the figure represent the numerical values of the given attributes. The data in the map is scaled to a range of 0 to 1, with brighter colors representing a value of 1 and darker colors representing a value of 0. The diagonal values are 1, indicating a perfect correlation between features. A reduction in values signifies a decrease in the correlation between features. This is helpful in diagnosing and predicting orthopedic diseases using the statistical analysis represented in the heatmap diagram. Figure 3 displays the box plot visualization for the dataset features classified using labeled analytics.

**Fig. 4:**
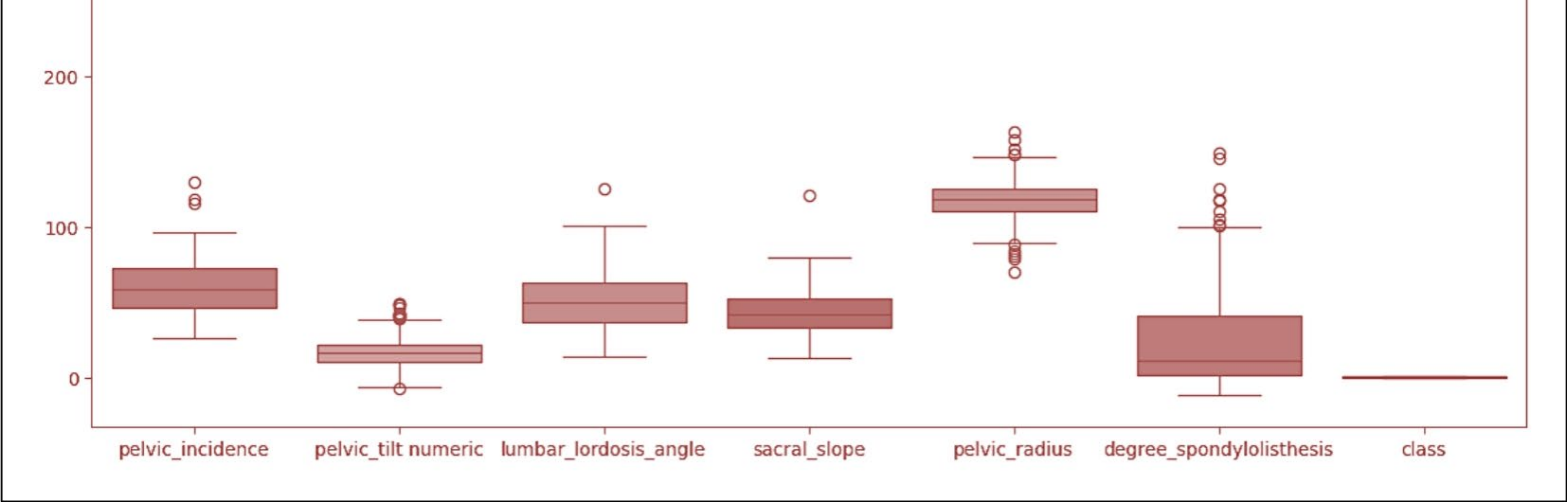
Boxplot visualization of dataset attributes.

Figure 4 displays a box plot that was used to analyze the distribution of the features. It is an excellent plot for illustrating the distribution of numerical data. You can use a box plot to visually represent the distribution of features in a dataset. We refer to this depiction as a box plot and use it to analyze the spatial distribution of features. We illustrate the six major enrollment features of the orthopedic dataset in this graph. Box plots divide the data into quartiles, with each piece representing approximately 25% of the dataset. Box plots are useful as they provide a visual context of the utilized data, enabling readers to quickly identify average values, the spread of the dataset, and inequality.

**Fig. 5:**
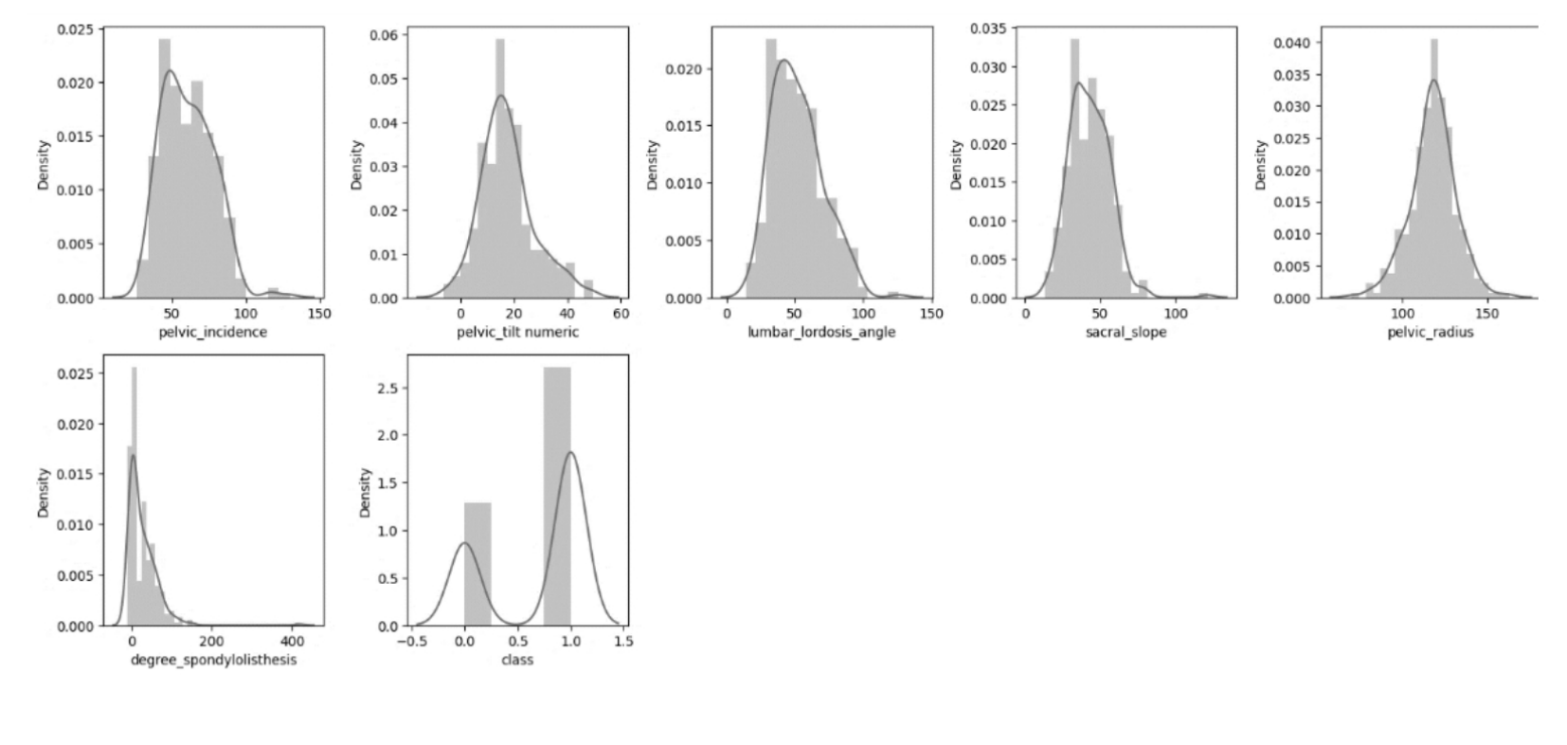
Histogram distribution analysis for the dataset features.

Figure 5 displays the distribution analysis of the characteristics. It visually represents the dataset’s statistical distribution by showing the frequency of data points at various intervals. This tool can be helpful for illustrating the distribution of the data that is represented as numbers. We analyzed the histogram of the features in this graphic, a typical graphing method used for displaying both continuous and discrete information collected on a scale consisting of intervals. It is commonly utilized to represent the fundamental characteristics of data distribution in a user-friendly manner. The selected features as shown in Table 4.

**Table 4:** Statistical of selected features.

Features	Num	Average	STD	Min	50%	Max
Pelvic_incidence	310.0	60.496653	17.236520	26.147921	58.691038	129.834041
Lumbar_lordosis_angle	310.0	51.930930	18.554064	14.000000	49.562398	125.742385
Pelvic_radius	310.0	117.920655	13.317377	70.082575	118.268178	163.071041
Degree_spondylolisthesis	310.0	26.296694	37.559027	-11.058179	11.767934	418.543082

### The proposed methodology

**Fig. 6:**
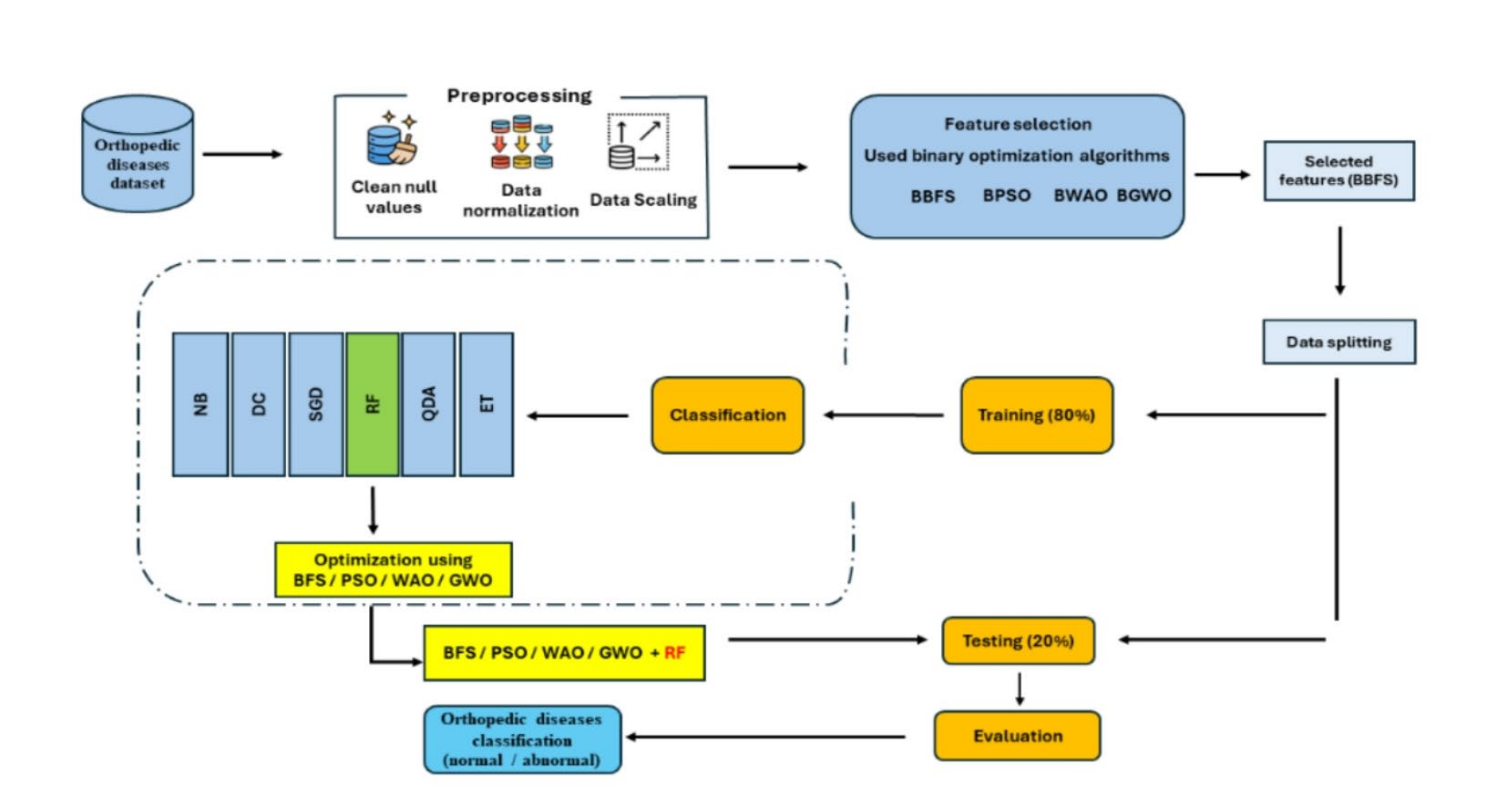
The optimized RF model based on BFS for Orthopedic’s disease classification.

This research employs a widely used dataset and a selection of algorithms with machine learning approaches for classifying patients in the field of orthopedics. Prior to evaluating the performance of a model, hyper-parameter optimization allows for precise adjustments. The study uses binary breadth-first search (BBFS), binary particle swarm optimization (BPSO), binary grey wolf optimizer (BGWO), and binary whale optimization algorithm (BWAO) for feature selections, and the BBFS makes an average less error, so we used BBFS as an optimal optimizer algorithm. Then six machine learning models, i.e., random forest (RF) classifier, stochastic gradient descent (SGD) classifier, Naïve Bayesian classifier (NBC), dummy classifier (DC), quadratic discriminant analysis (QDA) classifier, and extra trees (ET) classifier, were trained using a training set that was obtained through a feature selection optimizer (BBFS). Through experimentation, the RF model achieved the best results when compared with the others. The parameters of the RF model were optimized using four optimization algorithms: BFS, PSO, WAO, and GWO. The dataset used contains 310 instances and six distinct features. The results showed that the developed BFS-RF can improve the performance of the original classifier compared with other hybrid models. It was found that the BFS-RF performs better on the dataset. Figure 6 shows the optimized RF model based on BFS for Orthopedic’s disease classification (normal or abnormal).

Through the study, we exploit a shared dataset. to optimize the RF model using four optimization algorithms: BFS, PSO, WAO, and GWO, for classifying patients with Orthopedic’s disease. We use isolated parts of the whole dataset for training and testing targets. We can build classifier models using the training data. Afterwards, we evaluate the created models based on their ability to create a successful classification model for orthopedic illness. Random Forest is chosen using breadth-first search as the best method for adjusting variables. The first steps in creating a classification model for RF are determining the parameters that are predicted and the desired outcome. Next, try to tune the hyperparameter settings of the RF.

Eventually, the optimized Random Forest algorithm has been utilized for classification, and the model’s efficacy is assessed employing trial data. Experimental findings show that RF gave the highest accuracy of 91.4% before hyper-parameter adjustment, compared to 81.7%, 83.6%, 86.2%, 87.8.3%, and 89.3% for NB, DC, SGB, QDA, and ET, respectively. After applying hyperparameter tuning, the RF with BFS achieved 99.41% compared to 97.13%, 96.75%, and 93.95% for PSO-RF, WAO-RF, and GWO-RF, respectively. Therefore, it is an optimal method that utilizes the RF model for orthopedics’s illness categorization in contrast with other machine learning classifiers.

### Binary breadth-first search (BBFS)

BBFS is used in ML to identify the most relevant features from a dataset. It begins with considering all available features and iteratively removes the one that irrelevants and affects on the performance of the model and continues until a desired number of features remains. It simplifies the model, making it easier to train and comprehend. BBFS may help rectify overfitting, when the model performs well on training data but negatively on unknown data, by deleting unnecessary features. BBFS also speeds up training and improves model ability for generalization. BBFS has constraints like it takes much time for computations and especially for big datasets with several features. BBFS also depends on the performance metric, thus choose one that matches the model’s target. Finally, since BBFS eliminates features individually, it may overlook model-informing feature relationships.

A common feature selection approach in binary labeling scenarios is Binary Breadth-First Search (BBFS). The goal is to pinpoint and choose the most significant characteristics from a provided set of features in order to enhance the efficiency of a machine learning model. The algorithm utilizes Breadth-First Search (BFS) principles, a graph traversal technique, to effectively navigate the feature space. Algorithm 1 demonstrates the mathematical algorithm for binary breadth-first search used in feature selection.


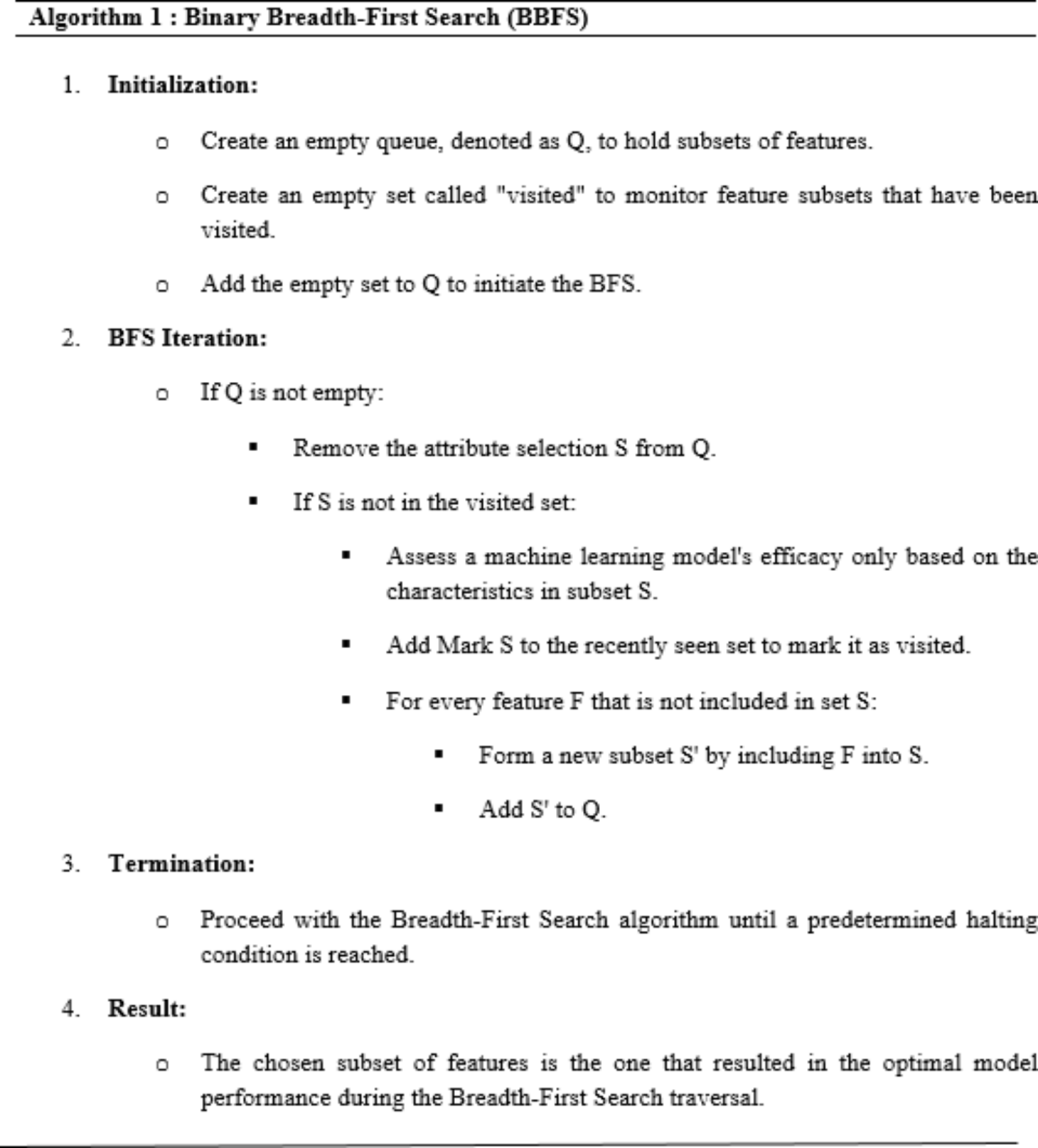


**Fig. 7:**
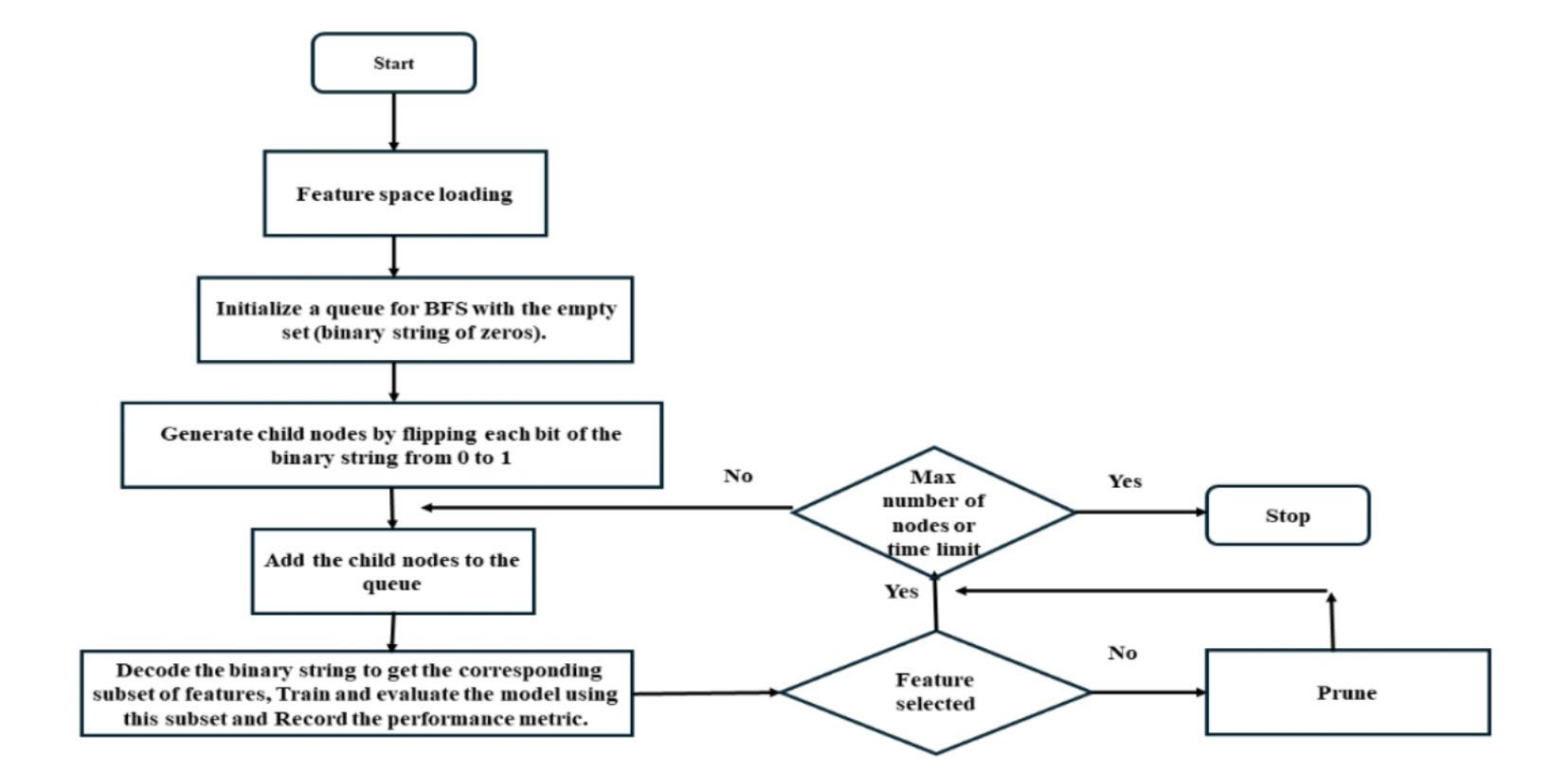
Encoding mechanism of BFS.

Algorithm 1 methodically examines the feature space in a breadth-first approach, guaranteeing that the chosen subset is based on the model’s performance. The halting criterion regulates the search space and prevents exhaustive exploration. Figure 7 displays the encoding mechanism of BFS.

### Machine learning models using hyperparameter optimization

The study presents an optimized RF model for orthopedic disease classifications, using BFS, PSO, WAO, and GWO algorithms to fine-tune hyperparameters. Biomechanical features from an orthopedic patient dataset^20^ were assessed for efficiency. BFS, PSO, WAO, and GWO are hyperparameter tuning methods that improve model accuracy by collecting observations with as much information as possible about the function and optimal value. The method efficiently investigates a wide variety of options by searching using different hyperparameter settings. algorithm 2 demonstrates the mathematical approach for BFS used in hyperparameter tuning.


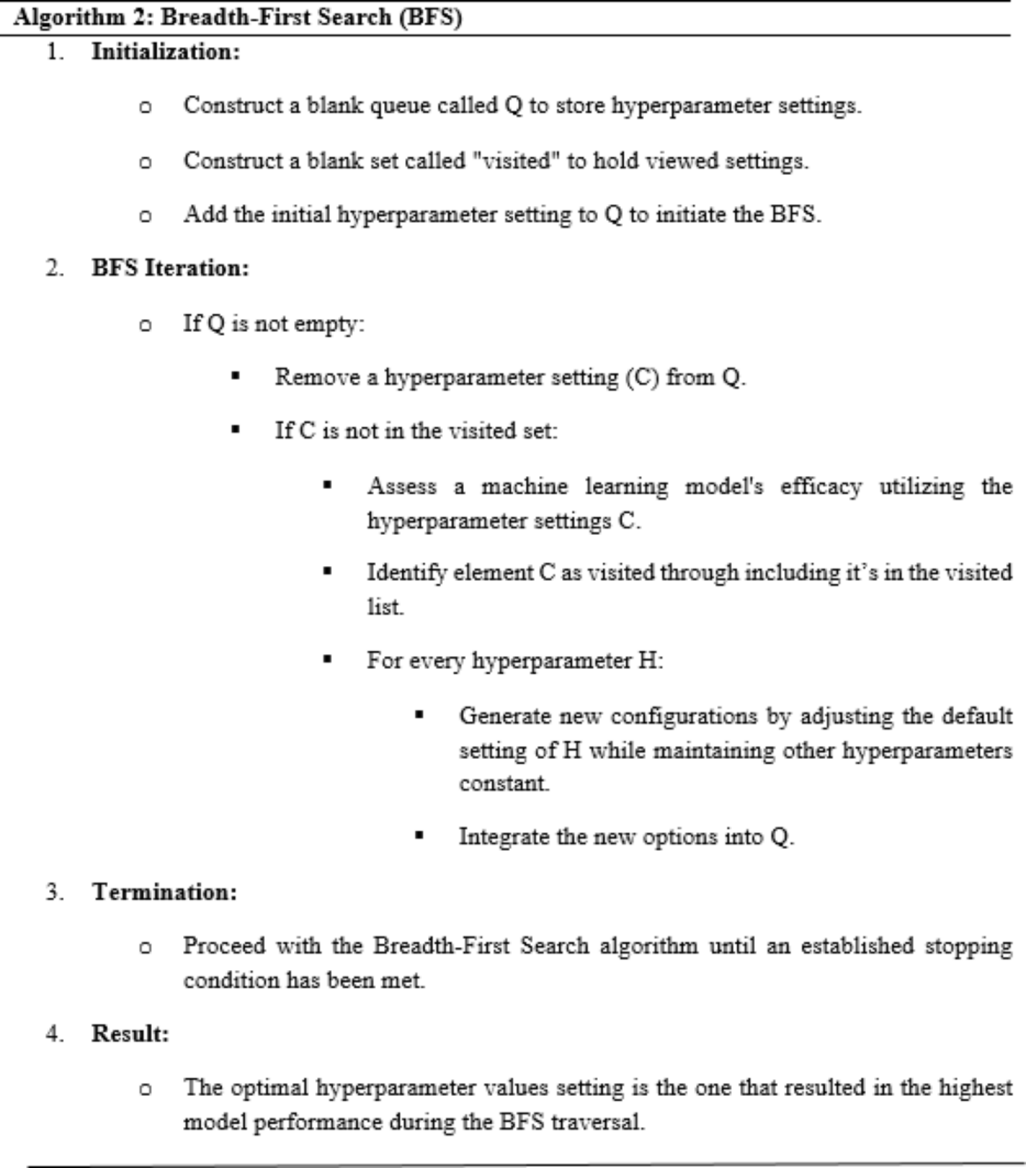


Algorithm 2 methodically examines the hyperparameter space, guaranteeing that different settings are assessed and compared using a breadth-first approach. The halting criterion regulates the search space to avoid exhausting investigation and enhance performance in adjusting hyperparameters.

## ML models

### Random forest

RF is an ensemble learning technique utilized for tasks involving regression as well as classification. It utilizes different classifiers to tackle intricate issues and enhance the performance of machine learning models. RF creates and merges decision trees to make predictions by utilizing methods such as majority voting or averaging the outputs. It decreases variability by utilizing a number of samples, random selections of characteristics, and aggregating predictions from smaller trees.

### Naive Bayes

NB is a probabilistic classifier that applies Bayes’ theorem under the assumption of feature independence. They excel at text classification tasks such as removing spam and sentiment detection. Various varieties of naive bayes include Gaussian naive bayes for continuous and normally distributed data, multinomial naive bayes for discrete features, and Bernoulli naive bayes for binary attributes. The selection of the option is contingent upon the characteristics of the data under examination.

### Dummy classifier

DC is a fundamental foundation classifier utilized in machine learning systems to assess the effectiveness of more sophisticated models. Its basic guidelines enable predictions, making it less complex than more sophisticated classifiers. Dummy classifiers are beneficial as they establish an initial performance that advanced models need to exceed. If a complex model fails to outperform a basic classifier, it indicates potential problems with either the algorithm or the dataset. They are beneficial in situations where there is an unequal distribution of classes, serving as a standard for evaluating more sophisticated methods.

### Stochastic gradient descent classifier

The SGD classifier is a linear regression technique that utilizes stochastic gradient descent for optimization. It is frequently utilized in large databases while prioritizing computing efficiency. Stochastic Gradient Descent (SGD) is adaptable and effective, particularly when working with extensive datasets and restricted processing capabilities. Achieving optimal results may necessitate a precise adjustment of hyperparameter settings such as rate of learning and normalization degree.

### Quadratic discriminant analysis classifier

QDA is a classification algorithm that categorizes data into various classes according to their characteristics. It is equivalent to linear discriminant analysis (LDA) but permits non-linear limits on decisions among classes. QDA offers greater flexibility by not assuming equal covariance matrices across classes, potentially enhancing performance. Estimating distinct matrices of covariance for each class might result in overfitting, particularly with high-dimensional data or a small number of training examples. Moreover, QDA may require significant processing resources, particularly when dealing with huge datasets. Although QDA has benefits, it necessitates a thoughtful evaluation of the balance between adaptability and overfitting, particularly in situations with high dimensionality or a limited sample size.

### Extra trees classifier

The ET, or Extremely Randomized Trees Classifier, is an ensemble learning technique that relies on DT. This is a modified version of the RF technique aimed at decreasing the model’s variance while keeping bias low. Extra trees add more unpredictability than RF, potentially resulting in enhanced performance under certain circumstances. The Extra Trees Classifier is a potent and effective method suitable for classification and regression applications, particularly when dealing with extended and complicated datasets.

#### Fitness function

Any optimized model depends on its fitness value to provide the best practice. In BFS-RF, the classification accuracy is selected via the search process as the solution attribute. With given parameters as the following:

$\:S$: A subset of features.

$\:{X}_{train}\left[S\right]$: Training data restricted to the features in subset *S.*

$\:{y}_{train\:}$: Corresponding labels for the training data.

$\:{X}_{val}\left[S\right]$: Validation data restricted to the features in subset *S.*

$\:{y}_{val}$: Corresponding labels for the validation data.

model: The machine learning model to be evaluated.

The fitness function *F(S)* can be defined in Eq. (3)

3 $$\:F\left(S\right)\:=\:\frac{1}{{n}_{val}}\:{\sum\:}_{i=1}^{{n}_{val}}I({\widehat{y}}_{i}=\:{y}_{i})$$

where $\:{\widehat{y}}_{i}$ is the predicted label for the $\:i-th$ validation sample using the model trained on *X*_train_ [*S*]. $\:{y}_{i}$ is the true label for the $\:i-th$ validation sample. $\:{n}_{val}$ is the number of samples in the validation set. $\:I(.)$ is the indicator function, which equals 1 if the condition inside is true (i.e., if the predicted label matches the true label) and 0 otherwise.

#### Data subsetting

Restrict the training and validation datasets to the features in subset S.

$$\:{X}_{train}\left[S\right]\:=\:{X}_{train}\left[:,S\right]$$

$$\:{X}_{val\:}\left[S\right]\:=\:{X}_{val}[:,S]$$

#### Model training

Train the machine learning model on the training data restricted to the features in subset *S.*

$$\:{\text{m}\text{o}\text{d}\text{e}\text{l}.\text{f}\text{i}\text{t}(X}_{train}\left[S\right],\:{y}_{train\:})$$

#### Prediction

Predict the labels for the validation data restricted to the features in subset S.

$$\:\widehat{y}=\:\text{m}\text{o}\text{d}\text{e}\text{l}.\text{p}\text{r}\text{e}\text{d}\text{i}\text{c}\text{t}\left({X}_{val}\:\right[S\left]\right)$$

#### Performance evaluation

Calculate the accuracy of the model using the predicted labels ŷ and the true labels $\:{y}_{val}$.

$$\:F\left(S\right)\:=\:\frac{1}{{n}_{val}}\:{\sum\:}_{i=1}^{{n}_{val}}I({\widehat{y}}_{i}=\:{y}_{i})$$

The relatively small sample of 310 incidents and 6 features limited the application of the findings to a general population with more diversified orthopedic problems. External validation tests the model on a new dataset not utilized for training. The study may be beneficial for a single problem but inaccurate for identifying many disorders. Table 5 shows the summary of the methods with their properties.

**Table 5:** Summary of the methods with their properties.

Method	Properties	Limitations
BBFS Feature Selection	Identifies relevant features for diagnosis	May not capture all important features, especially with limited data
BFS-Optimized Random Forest	High accuracy (99.41%)	Limited by dataset size and might not be disease specific

## Experimental setup

In this section, we depicted the experimental environment, followed by the interpretation of evaluation metrics in the proposed model.

### Setup

The ML models were run using the IDE Jupyter Notebook version 6.4.6. This software simplifies the process of developing and executing Python code. The application operates within a web-based browser and is compatible with other programming languages, such as Python version 3.8. The trial was executed on a machine equipped with an Intel Core i7 processor, 16GB of RAM, and the MS 10 operating system.

### Evaluation metrics

The study uses binary breadth-first search (BBFS), binary particle swarm optimization (BPSO), binary grey wolf optimizer (BGWO), and binary whale optimization algorithm (BWAO) for feature selections, and the BBFS makes less error. Then we apply six machine learning models, i.e., random forest (RF) classifier, stochastic gradient descent (SGD) classifier, Naïve Bayesian classifier (NBC), dummy classifier (DC), quadratic discriminant analysis (QDA) classifier, and extra trees (ET) classifier. Through experimentation, the RF model gave the best results, according to others, with the highest accuracy. The parameters of the RF model were optimized using four optimization algorithms: BFS, PSO, WAO, and GWO. Table 6 shows the configuration parameters of the optimizers. To check how well the optimized RF works on the dataset, this paper uses a number of prediction evaluation metrics, such as accuracy, sensitivity, specificity, F-score, and the AUC curve. Accuracy is calculated through the following Eq. (4):

4 $$\:Accuracy=\frac{TPos+TNeg}{TPos+FPos+FNeg+TNeg}$$

Sensitivity is the proportion of true positives among all actual positive cases (TP + FN), see Eq. (5).

5 $$\:Sensetivity=\frac{TP}{TP+FN}$$

6 $$\:Precision=\frac{TP}{TP+FP}$$

7 $$\:Specificity=\frac{TN}{TN+FP}$$

8 $$\:F-score=\frac{2\times\:Recall\times\:Precision}{Recall+Precision}$$

9 $$\:AUC=1/2\left(\frac{TP}{TP+FN}+\frac{TN}{TN+FP}\right)$$

**Table 6:** Configuration parameters for the optimization algorithms.

Algorithm	Parameter	Values
BFS	Number of vertices (*V*)	5
	Number of edges (*E*)	6
	Iterations	100
PSO	Acceleration constants	[5,5]
	Inertia Wmax, Wmin	[0.5, 0.8]
	Particles	50
	Iterations	100
WAO	Population size	200
	Iterations	100
GWO	Iterations Wolves	100 20

## Results

**Fig. 8:**
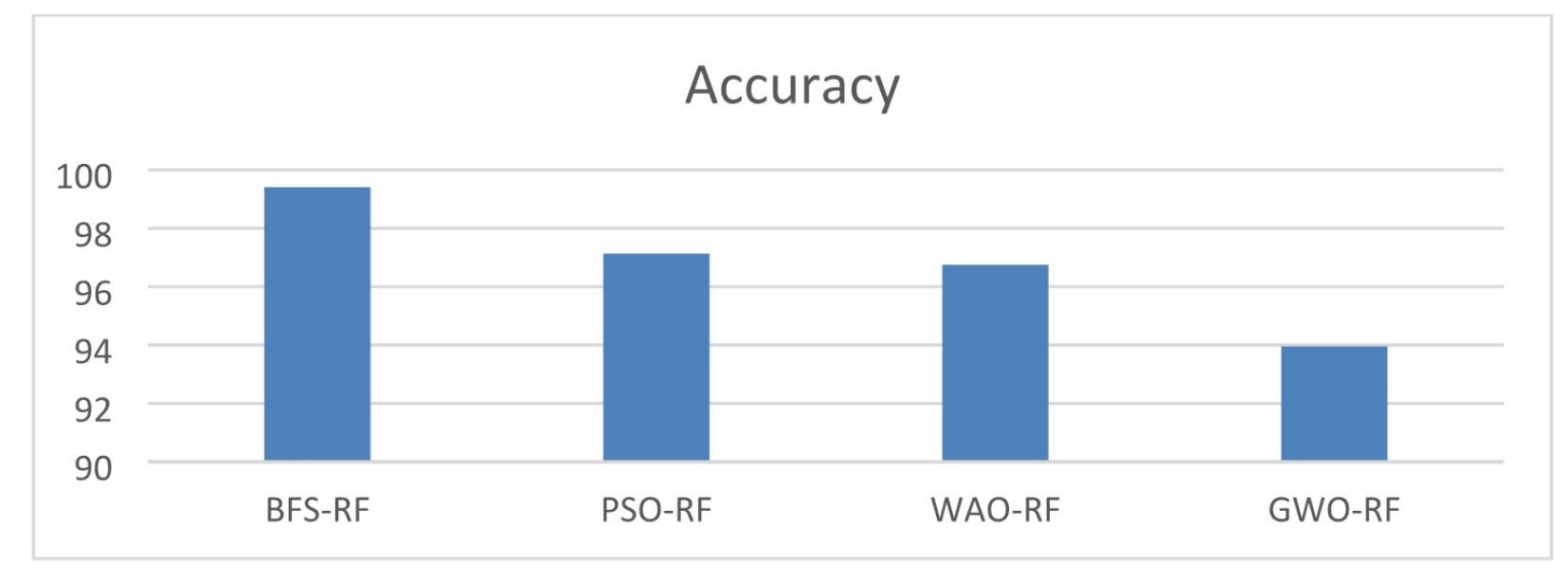
optimized RF model with accuracy using BFS, PSO, WAO, and GWO.

**Fig. 9:**
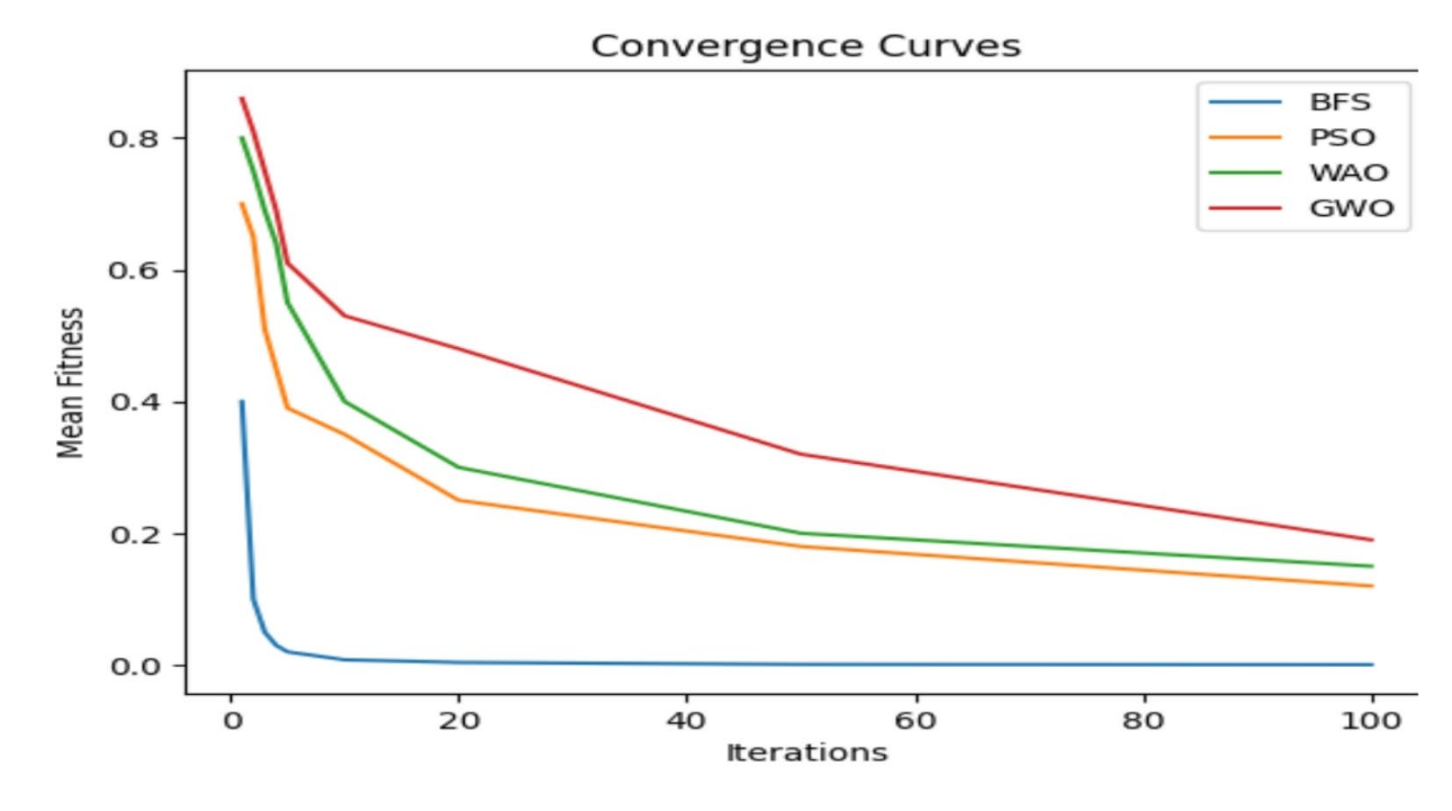
BFS convergence compared to others.

This section focuses on the outcomes of the experiments conducted to evaluate the performance of the proposed model. Table 7 shows the performance of an optimized RF model with preprocessing in terms of accuracy, sensitivity (TRP), specificity (TNP), F-score, the AUC curve, precision and fitted time. The optimized RF achieved high accuracy, sensitivity (TRP), specificity (TNP), F-score, the AUC curve, precision and fitted time is BFS-RF, with an accuracy of 99.41%, TRP of 99.42%, TNP of 99.41%, F-score of 99.42%, the AUC of 100%, precision of 99.41% and fitted time of 0.1152. The optimized RF model achieved the lowest result in GWO-RF, with an accuracy of 93.95%, a TRP of 93.95%, a TNP of 93.96%, an F-score of 93.95%, an AUC of 94.4%, precision of 93.96% and fitted time of 0.1328. Figure 8 represents the accuracy of the optimized RF model through optimizers (BFS, PSO, WAO, and GWO). Table 8 shows the performance of an optimized RF model without preprocessing in terms of accuracy, sensitivity (TRP), specificity (TNP), F-score, the AUC curve, precision and fitted time. Table 9 shows the RF parameters using BFS algorithm for the classification process. Figure 9 shows that BFS highlights high-speed convergence compared to others and from it we conclude in this specific optimization case, the BFS method has the highest rate of convergence and the lowest ultimate fitness value, therefore establishing its superior effectiveness. Both PSO and WOA algorithms exhibit strong performance, but with more delayed convergence and comparable end fitness values. Grey Wolf Optimizer (GWO), albeit having a slower convergence rate, may provide superior exploration capabilities, but at the cost of slower convergence and higher ultimate fitness values.

**Table 7:** Performance of the optimized RF using BFS, PSO, WAO, and GWO with preprocessing.

Optimized RF models	Accuracy	Sensitivity (TRP)	Specificity (TNP)	F-score	Precision	AUC	Fitted time
BFS-RF	99.41	99.42	99.41	99.42	99.41	100	0.1152
PSO-RF	97.13	96.93	96.94	96.93	96.93	97.20	0.1264
WAO-RF	96.75	96.76	96.75	96.76	96.75	97.08	0.1282
GWO-RF	93.95	93.95	93.96	93.95	93.96	94.72	0.1328

**Table 8:** Performance of the optimized RF using BFS, PSO, WAO, and GWO without preprocessing.

Optimized RF models	Accuracy	Sensitivity (TRP)	Specificity (TNP)	F-score	Precision	AUC	Fitted time
BFS-RF	96.03	95.74	95.52	95.5	95.72	96.06	0.3252
PSO-RF	93.698	93.18	93.15	93.08	93.19	93.39	0.3564
WAO-RF	92.98	92.93	92.93	92.97	92.83	93.45	0.3682
GWO-RF	90.563	90.26	90.05	90.26	90.1	91.41	0.3928

Table 10 displays the accuracy performance of the classification models without optimization, utilizing default settings. Utilizing initial values can streamline the modeling process by removing the necessity for manual adjustment of parameters. The built-in variables in the scikit-learn package are selected according to established best practices and have demonstrated effectiveness across several scenarios. The RF model achieved the highest accuracy of 91.4% among all models. The RF model optimized with BFS showed improvement, as seen in Table 7.

**Table 9:** Random Forest’s parameters using BFS algorithm for the classification process.

n_estimators	100
max_depth	5
min_samples_leaf	2
Bootstrap	True

**Table 10:** Performance of a model’s accuracy without optimizers.

Models	Accuracy
Naïve Bayesian Classifier (NBC)	81.7
Dummy Classifier (DC)	83.6
Stochastic Gradient Descent (SGD) Classifier	86.2
Quadratic Discriminant Analysis (QDA) Classifier	87.8
Extra Trees (ET) Classifier	89.3
Random Forest (RF) Classifier	91.4

Tables 11 and 12 show the analysis of variance (ANOVA) test results for BBFS and BFS-RF algorithm. Hyperparameter adjustment using Breadth-First Search (BFS) enhances the performance of the Random Forest (RF) model in comparison to the default values. Utilizing the BFS approach enhances hyperparameter optimization and leads to improved accuracy for the RF model. The analysis demonstrates notable disparities within groups for both BBFS and BFS-RF algorithms, suggesting that the treatments used exert a robust and statistically significant impact on their performance. The significant sum of squares for treatment suggests that the majority of the variability in the data can be accounted for by the various treatments, therefore proving their considerable impact. The methods yield robust findings, suggesting that the observed effects are not due to random chance but rather a consequence of the experimental settings. The attributes of ANOVA test illustrated as the following:

SS: The sum of squares shows how much variability is present within and between groups.

DF: The degrees of freedom help to determine the validity of the statistical test.

MS: Mean squares give an average measure of variation within and between groups.

F-Statistic: A higher F-value indicates greater variance between groups compared to within groups.

P-value: A very low p-value indicates that the observed differences are statistically significant.

**Table 11:** The analysis of variance (ANOVA) test results for BBFS algorithm.

	SS	DF	MS	F (DFn, DFd)	*P* value
Treatment (between columns)	0.1751	10	0.04023	F (10, 100) = 798.5	*P* < 0.0001
Residual (within columns)	0.000571	160	2.41E−05		
Total	0.1628	160			

**Table 12:** The analysis of variance (ANOVA) test results for BFS-RF algorithm.

	SS	DF	MS	F (DFn, DFd)	*P* value
Treatment (between columns)	0.01641	10	0.00517	F (10, 100) = 627.2	*P* < 0.0001
Residual (within columns)	0.000428	160	3.26E−06		
Total	0.03021	105			

**Fig. 10:**
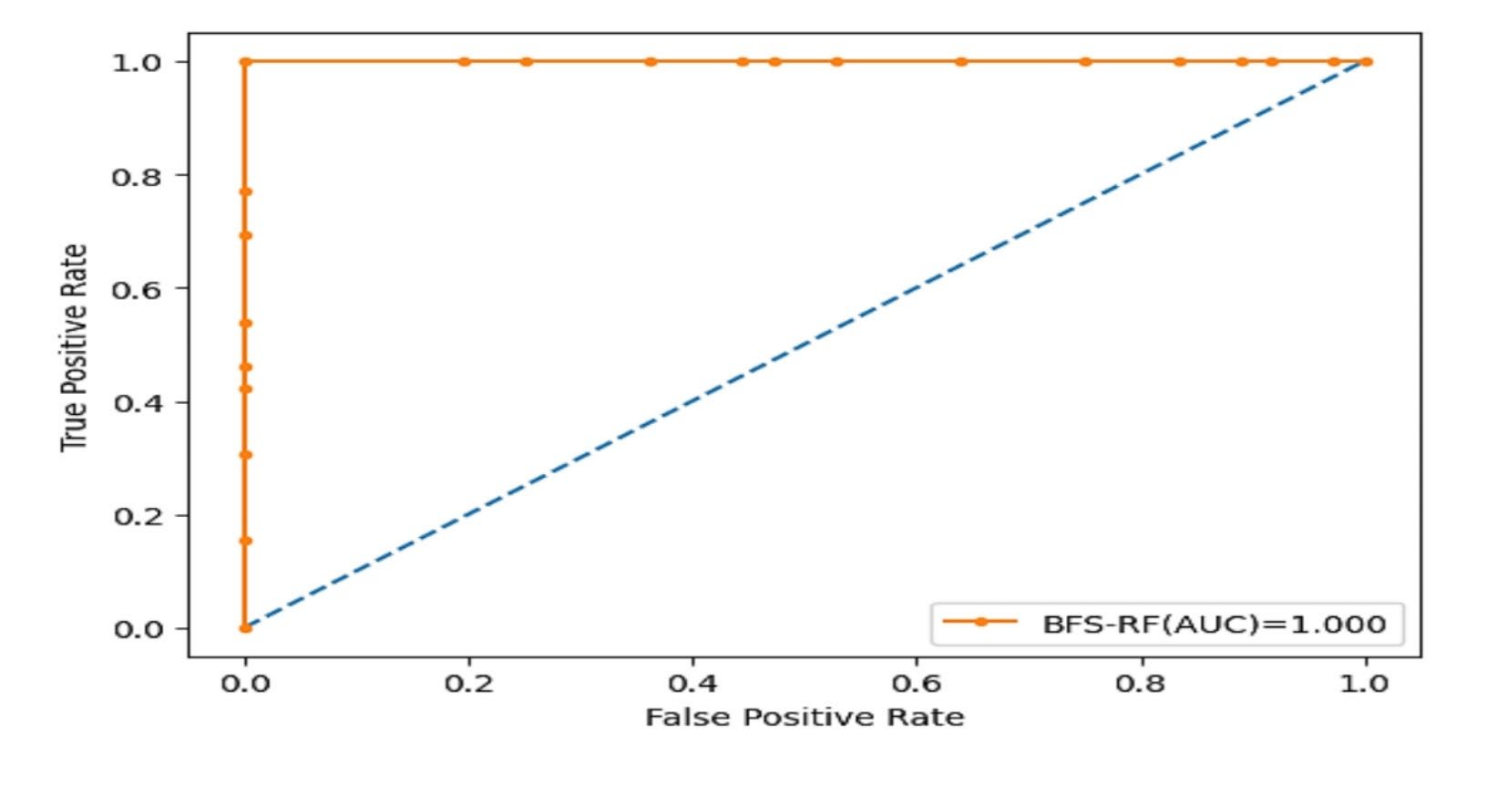
AUC curve for the BFS-RF model.

Figure 10 shows the AUC curve for the BFS-RF model using the dataset.

Table 13 depicted a comparison in the term of accuracy between the proposed BFS-RF model used in this study and other studies used the same dataset.

**Table 13:** Comparison between the proposed BFS-RF and other models used the same dataset in terms of accuracy.

Studies	Model Used	Accuracy
Proposed BFS-RF Model	Breadth-first search with random forest	**99.41%**
Elzeki et al. [23]	SVM	93.80%
Rubaiyat et al. [24]	Random forest	89%

## Conclusion and future work

An RF-optimized model based on BFS was developed for classifying orthopedic disease abnormal and normal people in this research. The dataset used contains 310 instances and six distinct features, and the target class was binary, with 1 indicating normal and 0 indicating abnormal. The data collecting phase guarantees the data is both comprehensive and well-structured. Effective management of noisy missing values is a substantial challenge, frequently arising from mistakes made during data recording. Two approaches are utilized: eliminating missing samples or rectifying null values by replacing them with the average value for each characteristic. The study uses binary breadth-first search (BBFS), binary particle swarm optimization (BPSO), binary grey wolf optimizer (BGWO), and binary whale optimization algorithm (BWAO) for feature selections, and the BBFS makes an average error of 47.29%. Then we apply six machine learning models, i.e., random forest (RF) classifier, stochastic gradient descent (SGD) classifier, Naïve Bayesian classifier (NBC), dummy classifier (DC), quadratic discriminant analysis (QDA) classifier, and extra trees (ET) classifier. Through experimentation, the RF model achieved the best results when compared with the others, with an accuracy of 91.4%. The parameters of the RF model were optimized using four optimization algorithms: BFS, PSO, WAO, and GWO, using a number of prediction evaluation metrics, such as accuracy, sensitivity, specificity, F-score, and the AUC curve. The results displayed that the optimized BFS-RF can enhance the performance of the standard classifier compared with other hybrid models. It was found that the BFS-RF performs better on the dataset with an accuracy of 99.41%. In the Future may involve extending the dataset utilized for categorizing orthopedic disease to include a more varied and diversified collection of people. Deep learning integrated methods could enhance accuracy and performance one of them, investigate various attribute selection approaches to determine the most crucial features for the classification. Validating the outcomes on a distinct set of data can enhance the robustness and generalizability of the model. Future research could focus on expanding the study to a broader population and exploring its possible application in a bigger healthcare network using the IoT and cloud computing systems. The model could test on diverse orthopedic illnesses. Optimizing Random Forest model interpretability to understand decision making. Integrating the model with healthcare applications and EHRs. The model will be tested on other medical applications^25–27^. Additionally, in the future, the proposed model can be integrated with these techniques^28–39^. This integration may potentially enhance the sophistication of orthopedic disease decision support systems, providing more nuanced and advanced capabilities.

## Electronic supplementary material

Below is the link to the electronic supplementary material.

Supplementary Material 1

## Data Availability

The data that support the findings of this study are available at https://www.kaggle.com/datasets/uciml/biomechanical-features-of-orthopedic-patients.

## References

[1] Blatter & Dvorak, J. Football for health - prevention is better than cure. *Scand. J. Med. Sci. Sports*. **20**, v–v (2010). 20210902 10.1111/j.1600-0838.2010.01114.x

[2] Srimani, P. & Koti, M. Medical diagnosis using ensemble classifiers—a novel machine-learning approach. *J. Adv. Comput.* (2013).

[3] Duan, Y. et al. Application and development of intelligent medicine in traditional Chinese medicine. *Curr. Med. Sci.*** 41**, 6 (2021). 10.1007/s11596-021-2483-2 PMC8654490 34881423

[4] Kim, D. et al. A data-driven artificial intelligence model for remote triage in the prehospital environment. *PloS One*. **13**, 10 (2018). 10.1371/journal.pone.0206006 PMC6198975 30352077

[5] Yao, L. H. et al. A novel deep learning–based system for triage in the emergency department using electronic medical records: retrospective cohort study. *J. Med. Internet. Res.*** 23**, 12 (2021). 10.2196/27008 PMC8749584 34958305

[6] Raita, Y. et al. Emergency department triage prediction of clinical outcomes using machine learning models. *Crit. Care*** 23** (2019). 10.1186/s13054-019-2351-7 PMC6387562 30795786

[7] Kwon, J. et al. Validation of deep-learning-based triage and acuity score using a large national dataset. *PloS One*. **13**, 10 (2018). 10.1371/journal.pone.0205836 PMC6188844 30321231

[8] Wang, W. et al. Attention mechanism-based deep learning method for hairline fracture detection in hand X-rays. *Neural Comput. Appl.*** 34**, 21 (2022). 10.1007/s00521-022-07412-0 PMC9244164 35789914

[9] Pranata, Y. et al. Deep learning and SURF for automated classification and detection of calcaneus fractures in CT images. *Comput. Methods Programs Biomed.*** 171** (2019). 10.1016/j.cmpb.2019.02.006 30902248

[10] Cheng, C. T. et al. A scalable physician-level deep learning algorithm detects universal trauma on pelvic radiographs. *Nat. Commun.*** 12**, 1 (2021). 33594071 10.1038/s41467-021-21311-3 PMC7887334

[11] Yaqoob, A. et al. Optimizing gene selection and cancer classification with hybrid sine cosine and Cuckoo Search Algorithm. *J. Med.Syst.*** 48**(1) (2024). 10.1007/s10916-023-02031-1 10.1007/s10916-023-02031-1 38193948

[12] Joshi, A. A. & Aziz, R. M. A two-phase Cuckoo Search based approach for gene selection and deep learning classification of cancer disease using gene expression data with a novel fitness function. multimedia tools and applications (Springer Science and Business Media LLC, 2024). 10.1007/s11042-024-18327-4

[13] Rahman, R. et al. Building resilient digital forensic frameworks for NoSQL database: Harnessing the blockchain and quantum technology. In* Sustainable Security Practices Using Blockchain, Quantum and Post-Quantum Technologies for Real Time Applications* 205–238 (Springer Nature Singapore, 2021). 10.1007/978-981-97-0088-2_11.

[14] Mahto, R. et al. A novel and innovative cancer classification framework through a consecutive utilization of hybrid feature selection. *BMC Bioinform*. **24**(1) (2023). 10.1186/s12859-023-05605-5. 10.1186/s12859-023-05605-5 PMC10724960 38102551

[15] Saxena, A. et al. A comprehensive evaluation of marine predator chaotic algorithm for feature selection of COVID-19. Evolving Systems (Springer Science and Business Media LLC, 2024). 10.1007/s12530-023-09557-2.

[16] Neggaz, N. et al. Boosting Manta Rays Foraging Optimizer by Trigonometry Operators: A Case Study on Medical Dataset. Neural Computing and Applications, vol. 36, no. 16 9405–9436 (Springer Science and Business Media LLC, 2024). 10.1007/s00521-024-09565-6.

[17] Houssein, E. H. et al. An efficient ECG arrhythmia classification method based on manta ray foraging optimization. In *Expert Systems With Applications*, vol. 181 115131 (Elsevier BV, 2021). 10.1016/j.eswa.2021.115131.

[18] Hashim, F. A. et al. Dimensionality reduction approach based on modified hunger games search: case study on Parkinson’s disease phonation. *Neural Comput. Appl.*** 35**(29), 21979–2005 (2023). 10.1007/s00521-023-08936-9.

[19] Hussain, K. et al. An efficient hybrid sine-cosine harris hawks optimization for low and high-dimensional feature selection. *Expert Syst. Appl.*** 176**, 114778 (2021). 10.1016/j.eswa.2021.114778.

[20] Biomechanical features of orthopedic patients (2024). https://www.kaggle.com/datasets/uciml/biomechanical-features-of-orthopedic-patients (accessed 11 Apr 2024).

[21] Islam Ayon, S., Milon Islam, M. & Diabetes prediction: A deep learning approach. *Int. J. Inf. Eng. Electron. Bus.*** 11**, 21–27 (2019).

[22] Pujianto, U., Wibawa, A. P. & Akbar, M. I. K-nearest neighbor (k-NN) based missing data imputation. In *2019 5th International Conference on Science in Information Technology (ICSITech)* (IEEE, 2019).

[23] Elzeki, O. et al. Biomedical healthcare system for orthopedic patients based on machine learning. *J. Eng. Appl.*** 16**, 616–622 (2006).

[24] Rubaiyat, N. et al. Classification and prediction of orthopedic disease based on lumber and pelvic state of patients. In *2019 IEEE International Conference on Electrical, Computer and Communication Technologies (ICECCT)*. (IEEE, 2019).

[25] Elshewey, A. M. et al. Bayesian optimization with support vector machine model for parkinson disease classification. *Sensors*** 23**(4), 2085 (2023). 10.3390/s23042085 PMC9961102 36850682

[26] Shams, M. Y. et al. A hybrid dipper throated optimization algorithm and particle swarm optimization (DTPSO) model for hepatocellular carcinoma (HCC) prediction. *Biomed. Signal Process. Control*. **85**, 104908 (2023).

[27] Tarek, Z. et al. An optimized model based on deep learning and gated recurrent unit for COVID-19 death prediction. *Biomimetics*. **8** (7), 552 (2023). 37999193 10.3390/biomimetics8070552 PMC10669113

[28] Shams, M. Y. et al. Water quality prediction using machine learning models based on grid search method. *Multimedia Tools Appl.*** 83** (12), 35307–35334 (2024).

[29] Tarek, Z. et al. Wind power prediction based on Machine Learning and Deep Learning models. *Comput. Mater. Continua*** 75**, 1 (2023).

[30] Alkhammash, E. H., Hadjouni, M. & Ahmed, M. Elshewey. A hybrid ensemble stacking model for gender voice recognition approach. *Electronics*** 11**(11), 1750. (2022).

[31] Eed, M. et al. Potato consumption forecasting based on a hybrid. *Stacked Deep Learn. Model. Potato Res.* 1–25 (2024).

[32] Abdelhamid, A. A. et al. Potato harvesting prediction using an Improved ResNet-59 model. *Potato Res.* 1–20 (2024).

[33] Alkhammash, E. H. et al. Optimized multivariate adaptive regression splines for predicting crude oil demand in Saudi Arabia. *Discrete Dyn. Nat. Soc.*** 2022** (1), 8412895 (2022).

[34] Alzakari, S. A. et al. Early detection of Potato Disease using an enhanced convolutional neural network-long short-term memory Deep Learning Model. *Potato Res.* 1–19. (2024).

[35] Alkhammash, E. H. et al. Application of machine learning to Predict COVID-19 spread via an optimized. *BPSO Model. Biomimetics*. **8** (6), 457 (2023). 37887588 10.3390/biomimetics8060457 PMC10604133

[36] Elshewey, A. M. et al. A Novel WD-SARIMAX model for temperature forecasting using daily delhi climate dataset. *Sustainability*** 15**(1), 757 (2022).

[37] Tarek, Z. et al. Soil erosion status prediction using a novel random forest model optimized by random search method. *Sustainability*** 15** (9), 7114 (2023).

[38] Elshewey, A. M. et al. Optimizing HCV disease prediction in Egypt: The hyOPTGB. *Framew. Diagn.*** 13** (22), 3439 (2023). 10.3390/diagnostics13223439 PMC10670002 37998575

[39] Alzakari, S. A. et al. An enhanced long short-term memory recurrent neural Network Deep Learning Model for Potato Price Prediction. *Potato Res.* 1–19 (2024).

