# Supplementary material for: Orthopedic disease classification based on breadth-first search algorithm
Source: Sci Rep. 2024 Oct 8;14:23368. doi: 10.1038/s41598-024-73559-6 (PMC11458584; doi:10.1038/s41598-024-73559-6)
Supplement: Supplementary file 1 — Supplementary Material 1 [file 41598_2024_73559_MOESM1_ESM.docx]

**Abbreviations**

BBFS Binary Breadth-First Search

BPSO Binary Particle Swarm Optimization

BGWO Binary Grey Wolf Optimizer

BWAO Binary Whale Optimization Algorithm

RF Random Forest

SGD Stochastic Gradient Descent

NBC Naive Bayes Classifier

DC Dummy Classifier

QDA Quadratic Discriminant Analysis

ET Extra Trees

BFS Breadth-First Search

PSO Particle Swarm Optimization

GWO Grey Wolf Optimizer

WAO Whale Optimization Algorithm

ML Machine Learning

KNN K-Nearest Neighbor

TPos True Positives

TP True Positives

FPos False Positives

FP False Positives
